# Defective expression of C20orf54 in esophageal dysplasia: a possible biomarker of esophageal carcinoma for early detection

**DOI:** 10.1186/s12957-022-02612-3

**Published:** 2022-05-12

**Authors:** Man Li, Jing Kong, Lianghai Wang, Hongjuan Yan, Weihua Liang, Ning Wang, Jin Zhao

**Affiliations:** 1 Department of Pathology, NHC Key Laboratory of Prevention and Treatment of Central Asia High Incidence Diseases, First Affiliated Hospital, Shihezi University School of Medicine, North 2 Road, Shihezi, Xinjiang, 832002 China; 2 Department of Pathology and Key Laboratory for Xinjiang Endemic and Ethnic Diseases, Shihezi University School of Medicine, North 2 Road, Shihezi, Xinjiang, China; 3 Department of Oncology, Pingyi County Hospital of Traditional Chinese Medicine, Pingyi, Shandong China; 4 Department of Stomatology, The First Affiliated Hospital to Shihezi University School of Medicine, Shihezi, Xinjiang, China

**Keywords:** *C20orf54*, Esophageal squamous cell carcinoma, Esophageal squamous intraepithelial neoplasia, Early detection, Precancerous lesions

## Abstract

**Background:**

*C20orf54* has been identified as an esophageal squamous cell carcinoma (ESCC) susceptibility gene in previous genome-wide association studies. Here, we attempted to clarify the expression level of C20orf54 in ESCC, non-tumoral esophageal tissues, and esophageal squamous intraepithelial neoplasia (ESIN).

**Methods:**

We assessed C20orf54 expression in 146 ESCC, 108 non-tumoral esophageal tissues, and 148 ESIN using immunohistochemistry on tissue microarrays. We also evaluated the possible correlations of C20orf54 expression with clinicopathological characteristics. The survival rates were analyzed using the Kaplan-Meier method and log-rank test.

**Results:**

C20orf54 expression was significantly lower in ESCC, high-grade ESIN, and low-grade ESIN than in the non-tumoral esophageal tissues. The level observed for ESCC was also significantly lower than that in low-grade ESIN and high-grade ESIN, whereas no difference was observed between high-grade ESIN and low-grade ESIN. Furthermore, the C20orf54 defective expression correlated significantly with differentiation, lymph node metastasis, and invasion depth. The overall survival time was inversely associated with lymph node metastasis, an advanced TNM stage (III + IV), and deeper invasion.

**Conclusions:**

This study provides the first evidence of C20orf54 defective expression in ESCC and precancerous lesions, demonstrating a potential role in tumor progression and metastasis. C20orf54 could be used as a potential biomarker for the early detection of ESCC.

## Introduction

Esophageal carcinoma (EC) ranks seventh among the causes of cancer-related mortality worldwide, and esophageal squamous cell carcinoma (ESCC) is the predominant histological type of EC in China [1]. The overall 5-year survival of ESCC averages 20%, and the best outcomes are associated with the disease diagnosed at the early stages [1, 2]. Identifying biomarkers and therapeutic targets in early detection remains an important task for novel diagnostic and therapeutic approaches. Esophageal squamous intraepithelial neoplasia (ESIN) has been regarded as a histologic precursor of ESCC [3] and has the clinical significance of on EC because it is encountered in cancerous esophagus frequently [4, 5]. Therefore, ESIN can be a reliable model for monitoring expression abnormalities and exploring squamous carcinogenesis at early stages.

*C20orf54*, known as human riboflavin transporter 2 (*hRFT2* or *SLC52A3*), encodes an open reading frame protein that can specifically and efficiently modulate riboflavin uptake. ESCC occurrence is a multistage process, and riboflavin deficiency can disrupt the integrity of the esophagus epithelium, similar to ESIN in humans [6]. Riboflavin supplements can lower ESCC incidence [7, 8] and reverse the esophageal hyperplasia to some extent [9]; however, the effects of intervention reveal individual differences that can be related to gene change. *C20orf54* has been identified as an ESCC susceptibility gene [7]; however, the expression of C20orf54 in ESCC is inconsistent. The gene is lower in ESCC tissues compared with normal tissues [10]. By contrast, Lu et al. [11] showed that the C20orf54 protein level in ESCC tissues was significantly higher than that in adjacent normal tissues. Several studies have also reported that C20orf54 plays important roles in the carcinogenesis and progression of other cancers, such as cervical [12] and gastric cancers [13].

The present study aims to investigate the association of C20orf54 protein expression with the susceptibility of different stages in ESCC development and attempts to explore the reason for the discrepant results. We assessed C20orf54 expression in 146 ESCC tissues, 108 non-tumoral esophageal tissues, and 148 ESIN using immunohistochemistry (IHC) by tissue microarrays (TMAs). We also evaluated the possible correlations of the expression with ESCC clinicopathological characteristics. We found that C20orf54 expression was downregulated during ESCC development and progression. Our findings suggest that C20orf54 defective expression in esophageal dysplasia can serve as a possible biomarker of EC for early detection.

## Materials and methods

### Patients and samples

A total of 146 ESCC formalin-fixed samples were obtained from the Department of Pathology, the First Affiliated Hospital, Shihezi University School of Medicine from 1997 to 2013. The study was approved by the Medical Ethics and Human Clinical Trial Committee of the Shihezi University School of Medicine. All recruited subjects were enrolled with written informed consent. No patients in this study had received prior surgery other than diagnostic biopsies, chemotherapy, or radiotherapy, and the clinical and pathological data were available for all patients. The TNM stage was evaluated according to the Cancer Staging Manual of 2017 [14]. The follow-up information was obtained through telephone calls or other methods. The follow-up deadline was the date of last contact or death. Patients who had received inadequate or less than 12 months of follow-up were excluded from this study. Additionally, 108 matched non-tumoral esophageal tissues, 94 low-grade ESIN (LGESIN) specimens, and 54 high-grade ESIN (HGESIN) specimens were also analyzed in this study.

### TMA construction

All paraffin-embedded samples were sectioned and stained for hematoxylin and eosin. Different regions of each sample were identified from the previous slides. The fields correspond to these selected regions were located in the remaining paraffin block for TMA construction. Tissue cylinders with a diameter of 1.0 mm were then punched from these areas of each donor tissue block and then inserted into a recipient paraffin block using a tissue arrayer (Alphelys, Plaisir, France). The area of each tissue cylinder was reviewed to ensure that at least 70% represented the specified region of interest in that sample. Finally, 4-μm-thick serial sections were prepared from the TMA blocks for IHC staining.

### IHC assay

IHC staining was performed using the Envision system (Dako, Carpinteria, CA, USA) to detect C20orf54 protein expression with TMA. Paraffin-embedded sections at 4-μm thick were baked at 65 °C for 30 min, then deparaffinized and rehydrated through graded alcohols. Antigen retrieval was then performed using an autoclave in citrate buffer solution buffer (pH 6.0) at 100 °C for 10 min, and then cooled to room temperature for approximately 40 min. Sections were then placed into the fresh methanol hydrogen peroxide (3%) for 10 min to remove endogenous peroxidase and then washed with tap water. After washing with phosphate-buffered saline (PBS), tissue sections were incubated with 150 μl C20orf54 antibody (1:400, sc-85426; Santa Cruz Biotechnology, Santa Cruz, USA) at 4 °C overnight, whereas negative control sections were incubated with PBS instead of the primary antibody. Epididymis tissues were recommended as a positive control according to instructions. The slides should warm to room temperature for 20 min and were then washed thrice in PBS and incubated with 150 μl of secondary antibody (Dako Cytomation Envision System, Dako, Denmark) for 30 min at 37 °C in an oven the following day. 3,3-Diamino-benzidine was then used to identify positive regions under the microscope (Olympus optical, Japan). Finally, these sections were stained lightly with hematoxylin to highlight the cell nuclei and then dehydrated with graded alcohol and xylene. Antifade mounting medium (ZSGB-Bio, Beijing, China) and glass coverslips were then added. Slides were stored at room temperature until analysis.

### Microscopic scoring of expression

The staining results of C20orf54 were positive when the brownish-yellow particles appeared in the cytoplasm. The sections were read, and the staining intensity and percentage of positive cells were calculated. The staining intensity was scored as 0 (no staining), 1 (weak), 2 (medium), and 3 (strong). The percentage of stained cells was categorized into the following: 0 (<5% stained cells), 1 (6–25% stained cells), 2 (26–50% stained cells), 3 (51–75% stained cells), and 4 (76% stained cells or larger). The product of both scores was used to identify four categories of expression: +++ (9–12), ++ (5–8), + (2–4) and −(0–1) [15]. The staining results were evaluated by two pathologists who were blinded to the clinicopathologic data, and the third pathologist scored the section when the two experts disagreed with each other to determine the immunohistochemical results.

### Statistical analysis

Statistical Package for the Social Science (SPSS) (IBM Corp, Armonk, NY, USA) version 17.0 was used for the statistical analyses. The *χ*^2^ tests were used to determine the statistical significance of C20orf54 protein expression in the ESCC and ESIN samples compared with the non-tumoral control tissues, as well as the correlations between C20orf54 expression and clinicopathologic factors. The survival rates were calculated using the Kaplan-Meier method, whereas the survival curves were analyzed using the log-rank test. Receiver operating characteristic (ROC) curves and the area under the ROC curve (AUC) were used to evaluate the specificity and sensitivity of ESCC and ESIN prediction. The univariate and multivariate hazard ratios for different variables were calculated using the Cox proportional hazards model. A *P*-value of less than 0.05 was considered statistically significant in all statistical analyses.

## Results

### Clinicopathologic demographics of the ESCC patients

The present study included 146 patients with ESCC, of which 104 (71.23%) were men and 42 (28.77%) were women. The patient ages at the time of surgery ranged from 36 years to 81 years, with a median age of 62 years. The proportion of smokers among ESCC patients was 99 (67.81%), whereas 47 (32.19%) were non-smokers. The proportions of drinkers and non-drinkers were 82 (56.16%) and 64 (43.84%), respectively. Of the 146 ESCC cases, 19 (13.01%) were well differentiated, 83 (55.48%) were moderately differentiated, and 44 (30.14%) were poorly differentiated [16]. Lymph node metastasis was present in 64 (43.84%) of the patients. Furthermore, 94 (64.38%) of the patients were at the I or II clinical stage, whereas 52 (35.62%) were at stage III or IV. The proportions of the upper, middle, and lower tumor locations were 15 (10.27%), 80 (54.80%), and 51 (34.93%), respectively. The invasion depth shows that 6 (4.11%) were Tis and T1, whereas 140 (95.89%) were T2, T3, and T4.

### C20orf54 protein expression decreased in ESCC and ESIN compared with the non-tumoral squamous epithelium tissue

**Fig. 1:**
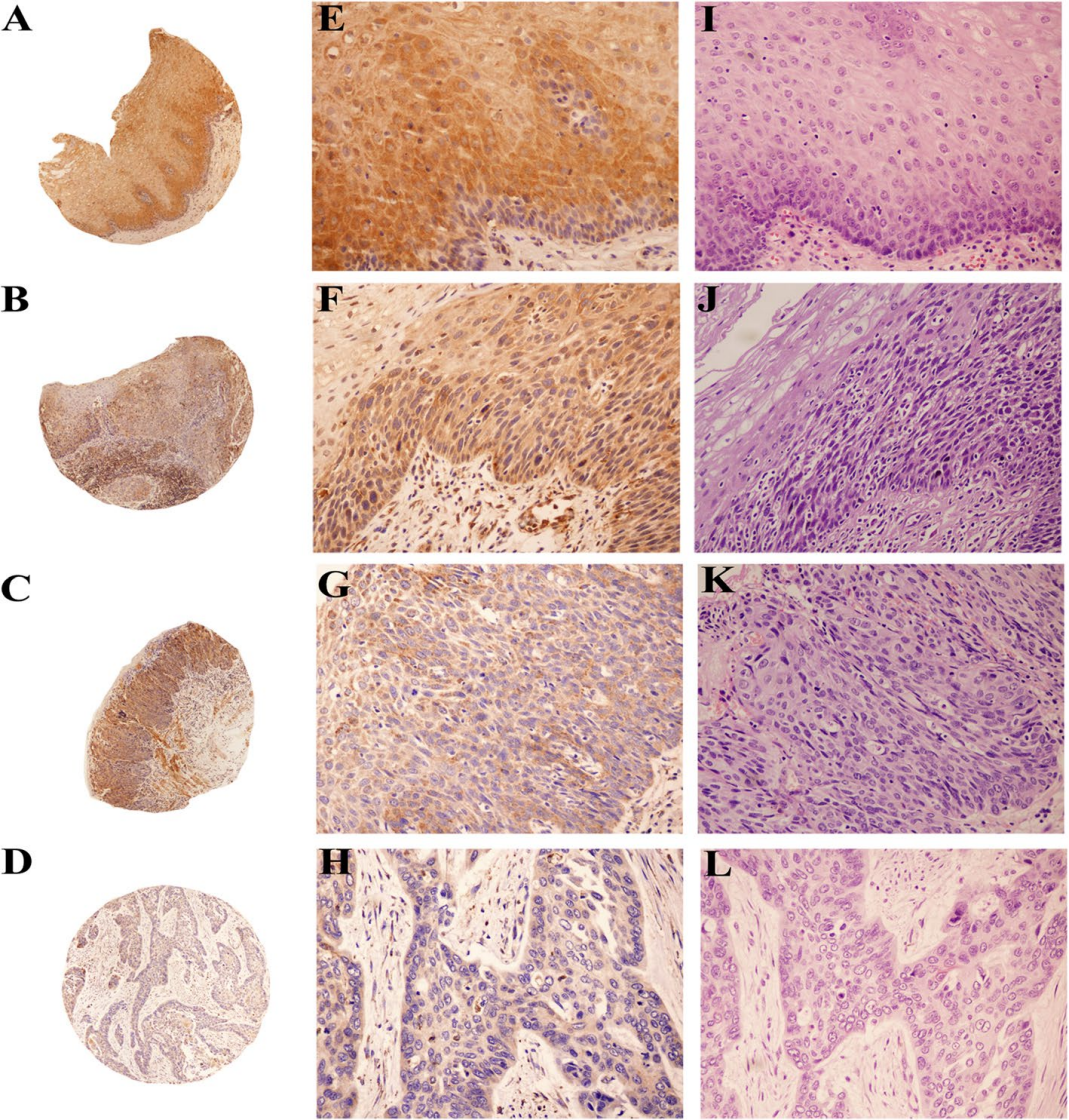
Representative images of the IHC staining of C20orf54 protein expression, as well as hematoxylin and eosin staining, in different esophageal lesion tissues. C20orf54 primarily expressed in the cytoplasm (original magnification, **A**–**D**: ×40, **E**–**L**: ×200). **A**, **E** Strong staining of C20orf54 in non-tumoral esophageal tissue. **B**, **F** Moderate staining of C20orf54 in LGESIN. **C**, **G** Weak staining of C20orf54 in HGESIN. **D**, **H** C20orf54 staining is negative in ESCC. Hematoxylin and eosin staining is shown for non-tumoral esophageal tissue (**I**), LGESIN (**J**), HGESIN (**K**), and ESCC (**L**)

**Fig. 2:**
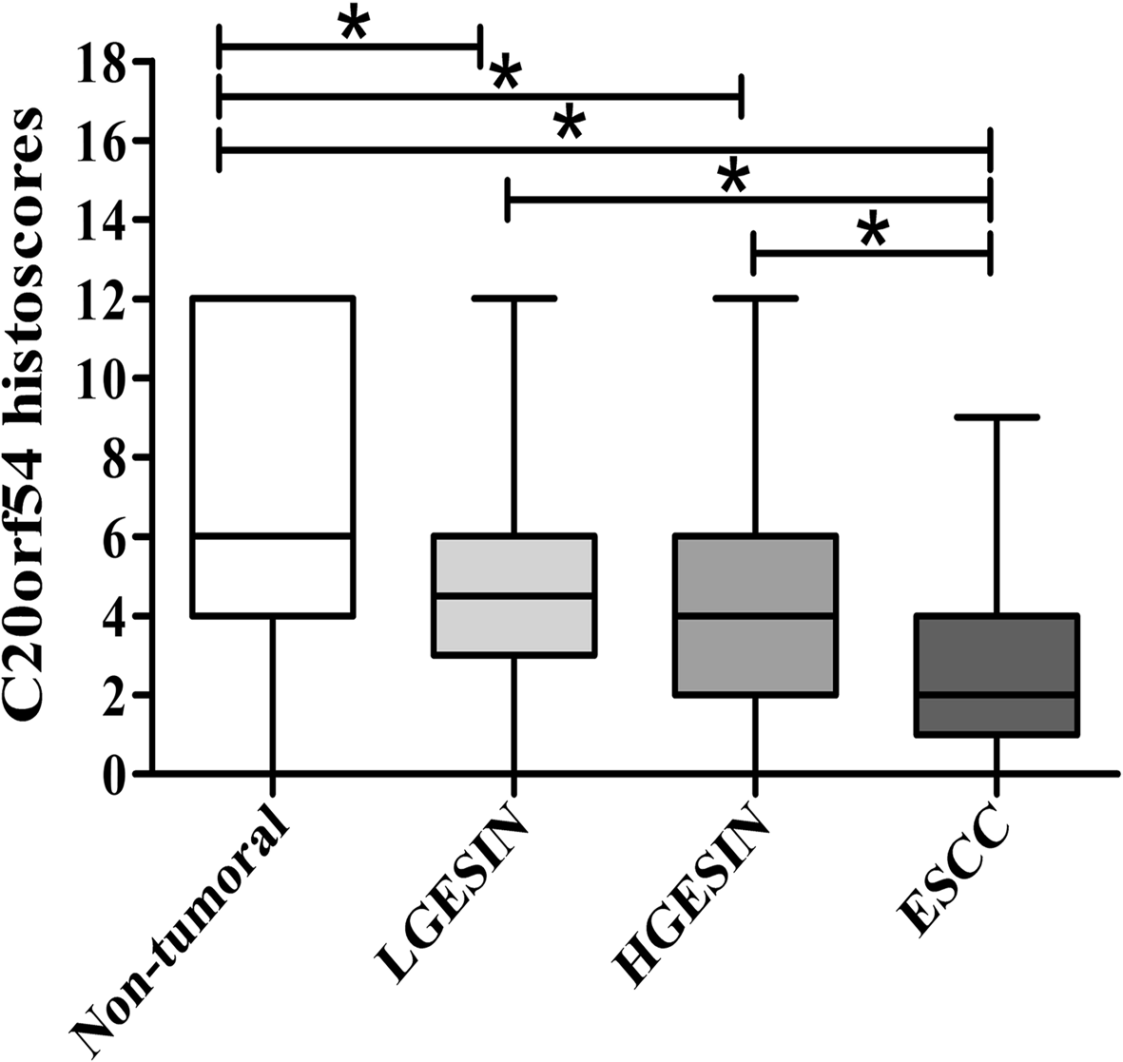
Expression levels of C20orf54 in different esophageal lesion tissues. The box plot shows significantly higher expression of C20orf54 in non-tumoral esophageal tissues than that in LGESIN, HGESIN, and ESCC tissues. The C20orf54 expression was significantly lower in ESCC than in LGESIN and HGESIN (*P < 0.001)

A total of 108 non-tumoral control tissues, 94 LGESIN, 54 HGESIN, and 146 primary ESCC tissue samples were used in the present study. IHC staining demonstrates that C20orf54 was localized in the cytoplasm (Fig. 1). The distributions of the staining patterns of C20orf54 were significantly different among these four groups (Table 1). We observed that the rate of C20orf54 expression had a decreasing tendency as the cell type progressed from normal (non-tumoral) to increasing levels of metastasis (LGESIN followed by HGESIN and ESCC). Positive staining for C20orf54 was generally observed within the non-tumoral control tissues, but weak or no C20orf54 staining was mostly detected in LGESIN, HGESIN, and ESCC tissues. Approximately, 74.07% (80 of 108) of the control cells was highly stained (strong staining and medium staining) for C20orf54, whereas the value sharply decreased to 45.74% (43 of 94), 33.33% (18 of 54), and 11.64% (17 of 146) in LGESIN, HGESIN, and ESCC tissues, respectively (both *P <* 0.05 compared with the non-tumoral control tissues). The positive rate in ESCC was the least among the four groups, and the value was significant (*P <* 0.05) (Fig. 2).

**Table 1:** Expression of C20orf54 in non-tumoral, ESCC, LGESIN and HGESIN tissues

Group	n	C20orf54 expression (%)				*P* value
		-	+	++	+++	
Non-tumoral	108	2 (1.85)	26 (24.07)	35 (32.40)	45 (41.67)	< 0.001^*^
LGESIN	94	6 (6.38)	45 (47.87)	33 (35.1)	10 (10.63)	
HGESIN	54	9 (16.67)	27 (50)	15 (27.78)	3 (5.56)	
ESCC	146	38 (26.02)	91 (62.33)	14 (9.59)	3 (2.05)

### Association of C20orf54 protein expression in ESCC tissues with clinicopathological characteristics

We also evaluated the relationship between the C20orf54 expression and clinicopathological characteristics of ESCC, including age, gender, histological grade, lymph node metastasis status, TNM stage, tumor location, invasion depth, smoking status, and drinking status. Notably, we can infer that the C20orf54 defective expression in tumor cells appears to be significantly associated with poor differentiation (*P* = 0.014), lymph node metastasis (*P* = 0.021), and invasion depth (*P* = 0.019). These data suggest that the downregulated expression of C20orf54 can be involved in tumor metastasis and aggressiveness and contribute to tumor development. However, the other parameters such as age, gender, TNM stage, tumor location, smoking status, and drinking status had no significant relationship with C20orf54 expression (Table 2).

**Table 2:** The correlation between C20orf54 protein expression and clinicopathologic factors in ESCC

Characteristics	*n*	C20orf54 protein expression (%)		*P* value
		−/+	++/+++	
Age (years)^a^				
< 62	56	49 (87.5)	7 (12.5)	0.799
≥62	90	80 (88.89)	10 (11.11)	
Gender				
Male	104	93 (89.42)	11 (10.58)	0.728
Female	42	36 (85.71)	6 (14.29)	
Differentiation^b^				
Well	32	28 (87.50)	4 (12.50)	0.014^*^
Moderate	84	71 (84.53)	13 (15.48)	
Poor	30	30 (100)	0	
L/N metastasis				
Negative	82	68 (82.93)	14 (17.07)	0.021^*^
Positive	64	61 (95.31)	4 (4.69)	
TNM stage^c^				
I/II	95	83 (87.37)	12 (12.63)	0.612
III/IV	51	46 (90.20)	5 (9.80)	
Tumor location				
Upper + middle	94	85 (90.43)	9 (9.57)	0.295
Lower	52	44 (84.62)	8 (15.38)	
Invasion depth				
Tis,T1	6	3 (50)	3 (50)	0.019^*^
T2,T3,T4	140	126 (90)	14 (10)	
Smoking status				
Non-smokers	60	52 (86.67)	8 (13.33)	0.595
Smokers	86	77 (89.53)	9 (10.47)	
Drinking status				
Non-drinkers	82	71 (86.58)	11 (13.42)	0.450
Drinkers	64	58 (90.63)	6 (9.37)	

*L/N* lymph node metastasis. ^a^Mean age. ^b^Histologic grade was based on a World Health Organization classification published in 2019 [16]. ^c^TNM stage was based on the American Joint Committee on Cancer Q1 criteria published in 2017 [14]

### C20orf54 can be a potential diagnostic biomarker of ESCC and ESIN

**Fig. 3:**
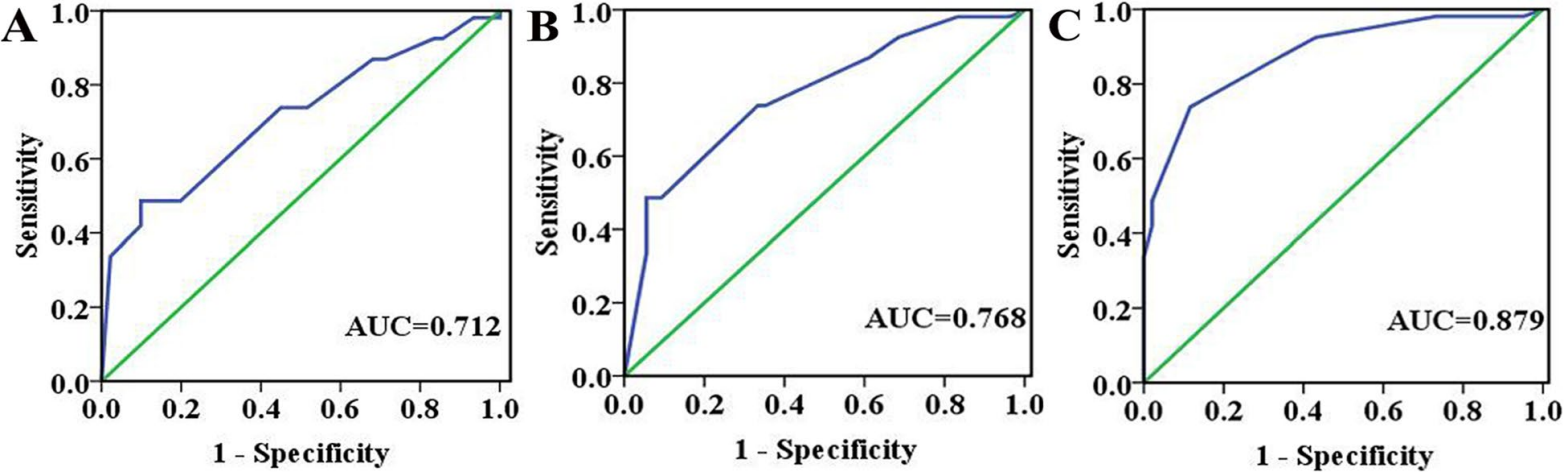
ROC curve analysis of the C20orf54 IHC scores for detecting LGESIN (**A**), HGESIN (**B**), and ESCC (**C**) tissues from the controls

We then used ROC curves to evaluate the C20orf54 IHC scores determined for each of the ESCC, HGESIN, LGESIN, and control tissues. We found that the ESCC, HGESIN, and LGESIN tissues were easily distinguished from the controls with ROC AUC values of 0.879 (95% CI, 0.835–0.922), 0.770 (95% CI, 0.693–0.843), and 0.710 (95% CI, 0.638–0.780), respectively (Fig. 3). We next used cutoff levels to determine the sensitivity and specificity values for each cancerous tissue type that optimized the diagnostic accuracy rate, as well as minimized the false-negative and false-positive rates (Table 3). The sensitivity and specificity values for ESCC were 74.1% and 88.4% (cutoff score of 5), whereas HGESIN and LGESIN were 49.1% and 94.4% (cutoff score of 7.5), and 49.1% and 89.4% (cutoff score of 7.75), respectively. Thus, these results support the conclusion that C20orf54 can be a potential diagnostic biomarker for ESCC and ESIN.

**Table 3:** High sensitivity, specificity, and AUC values of C20orf54 in ESCC, HGESIN, and LGESIN

Comparison	AUC	Sensitivity (%)	Specificity (%)	Cutoff value^a^
ESCC vs control	0.879	0.741	0.884	5
HGESIN vs control	0.770	0.491	0.944	7.5
LGESIN vs control	0.710	0.491	0.894	7.75

^a^Cutoff level was set to provide optimal sensitivity and specificity

### C20orf54 defective expression predicts poor prognosis in ESCC

**Fig. 4:**
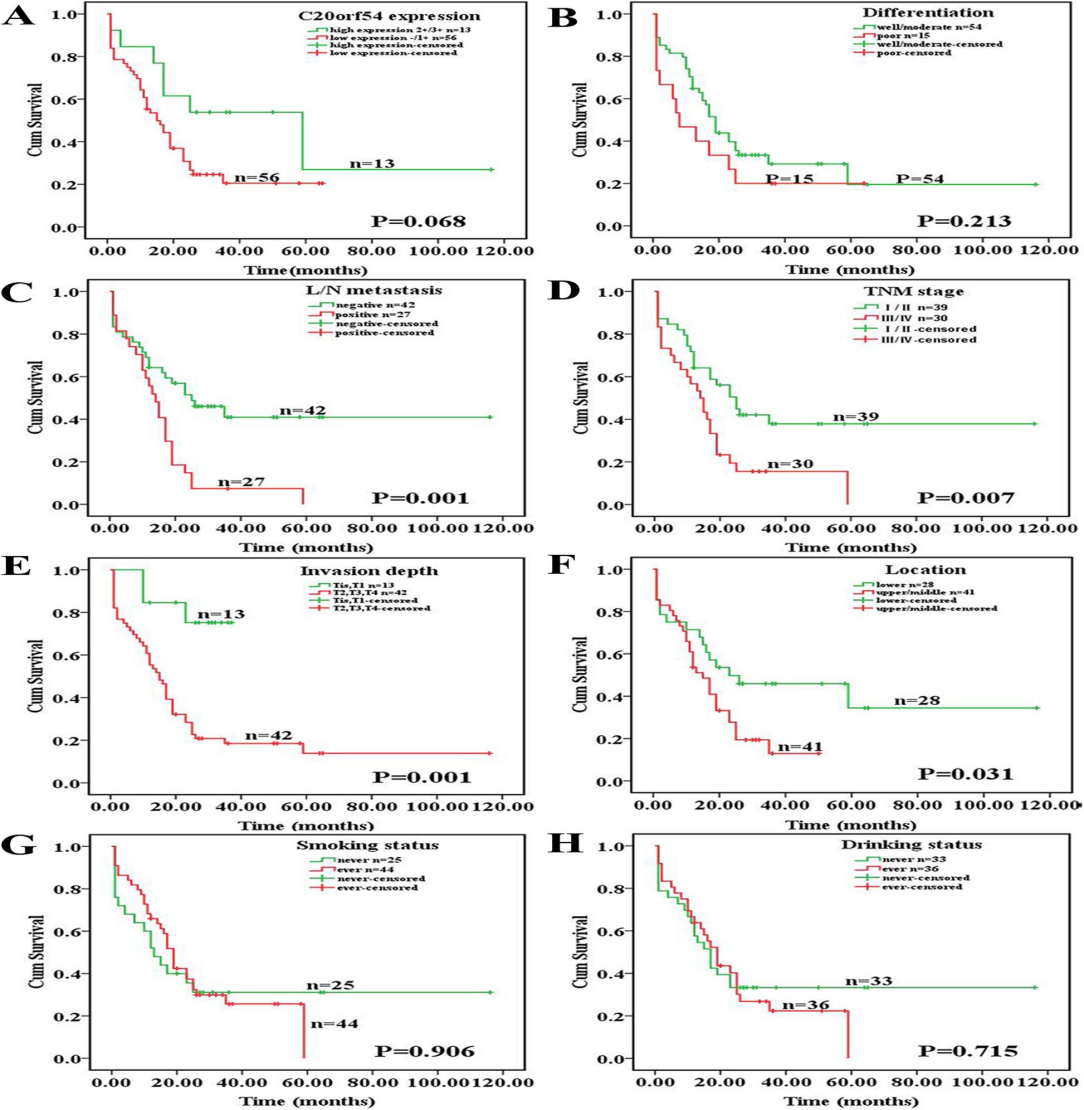
Survival analyses for C20orf54 expression and other clinical parameters in ESCC patients. The Kaplan-Meier survival curves that show the association among ESCC patient survival times with the C20orf54 expression level (**A**, *P* = 0.068), differentiation (**B**, *P* = 0.213), L/N metastasis (**C**, *P* = 0.001), TNM stage (**D**, *P* = 0.007), invasion depth (**E**, *P* = 0.001), location (**F**, *P* = 0.031), smoking status (**G**, *P* = 0.906), and drinking status (**H**, *P* = 0.715)

We examined the correlation between the C20orf54 expression and postoperative survival of patients who underwent curative surgery, and parameters including differentiation, lymph node metastasis, invasion depth, TNM stage, tumor location, smoking status, and drinking status. The mean follow-up period of postoperative survival was 20 months, ranging from 1 to 116 months after treatment. Furthermore, we found no difference in survival time using the Kaplan-Meier analysis. Thus, we found a tendency of patients who expressed higher C20orf54 to experience a longer overall survival time than patients with lower C20orf54 expression (Fig. 4A). Moreover, the postoperative mortality rate was higher in patients with low C20orf54 expressed than in those with high expression. Other parameters, such as lymph node metastasis, invasion depth, TNM stage, and tumor location, showed significant differences in survival time (Fig. 4, both *P* < 0.05).

Finally, a multivariate Cox regression analysis was performed for the C20orf54 expression, and all clinicopathological factors were included in the univariate analysis to explore whether additional prognostic information was involved in the IHC analysis. Statistically, the C20orf54 detective expression was an insignificant factor in the multivariate Cox regression model. These data generally indicate that C20orf54 expression was an insignificant independent prognostic factor for poor prognosis in ESCC, but it was unsuitable for TNM stage and invasion depth (P = 0.040, HR = 2.519; P = 0.009, HR = 5.817, Table 4).

**Table 4:** Univariate and multivariate survival analyses of clinicopathological characteristics

Characteristics	Univariate analysis		Multivariate analysis	
	HR (95% CI)	*P* value	HR (95% CI)	*P* value
C20orf54 defective expression	2.044 (0.912,4.583)	0.082	1.522 (0.642, 3.608)	0.340
Differentiation^a^ (poor)	1.490 (0.775,2.865)	0.232	1.256 (0.599, 2.635)	0.546
TNM^b^ (III+IV)	2.108 (1.190,3.733)	0.011^*^	2.519 (1.043, 6.082)	0.040^*^
L/N metastasis (positive)	2.429 (1.369,4.308)	0.002^*^	0.910 (0.368, 2.247)	0.838
Invasion depth (T2, T3, T4)	5.24 (1.626,16.935)	0.006^*^	5.817 (1.542, 21.94)	0.009^*^
Location (upper, middle)	1.906 (1.029,3.531)	0.040^*^	1.859 (0.970, 3.564)	0.062
Smokers (ever)	0.966 (0.534,1.748)	0.909	0.93 (0.437, 1.988)	0.856
Drinkers (ever)	1.107 (0.629,1.949)	0.724	0.84 (0.436, 1.768)	0.715

*L/N* lymph node metastasis, *HR* hazard radio, *CI* confidence interval of the estimated HR. ^a^Histologic grade was based on a World Health Organization classification published in 2019 [16]. ^b^TNM stage was based on the American Joint Committee on Cancer Q1 criteria published in 2017 [14]

## Discussion

Most recently, C20orf54 was identified as a susceptibility gene for ESCC in Chinese Han populations [7]. However, limited information is available in C20orf54 expression studies, and the two documented reports show conflicting results in this regard [10, 11]. Conclusive results are needed to demonstrate the associations of C20orf54 with ESCC at the expression level. The present study demonstrates that the defective expression of the C20orf54 protein was significantly associated with the ESCC development and progression. To the best of our knowledge, this study is the first to demonstrate that C20orf54 expression decreased during the ESCC developing process of ESCC. This scenario suggests that C20orf54 may play an important role in ESCC and its related precancerous lesions.

Riboflavin is mainly used in the complete protection of esophageal mucosal epithelial to prevent lesions; riboflavin supplements can lower the incidence of ESCC [7, 8] and reverse esophageal hyperplasia [13]. C20orf54 protein used as the specific transporter can likely have a certain relationship with the ESCC development. The present study shows that LGESIN, HGESIN, and ESCC tissues exhibited significantly lower C20orf54 expression compared with non-tumoral tissues. HGESIN and ESCC expressed C20orf54 at a lower level than LGESIN. Other studies [13] found that the riboflavin level in ESIN was higher than in ESCC, but lower than the level in normal esophageal tissues; this condition was consistent with our protein results. Machado et al. [17] found that riboflavin inhibits melanoma invasion through the negative-regulated Hedgehog pathway and downregulates GLI-1 and PTCH-1 expression, which indicates that C20orf54 may be involved in ESCC tumorigenesis through this pathway. However, further explorations are required to verify this hypothesis, such as detecting the expression of key molecules on the Hedgehog pathway, correlation analysis, and several cytological functional tests.

Our observations of downregulated protein expression of C20orf54 in ESCC tissues are in agreement with the findings by Aili et al. [12], but differ from those by Jiang et al. [11], who found an opposite result in C20orf54 between ESCC and matching adjacent normal tissues. This discrepancy may result from several factors, of which sample size may be one as Jiang et al. used a smaller sample size (*n* = 26) [11] than those by Aili et al. (*n* = 65) [12] and by ours in this study (*n* = 146). The other possible factors resulting in the discrepancy may be population heterogeneity, environmental factors, and diet habits of the people, which need to be further clarified by a more detailed analysis of the stratified analysis and larger sample size.

Gene expression is modulated by both genetic and epigenetic mechanisms [18]. Svetlana et al. [19] found that missense mutations in hRFT-2 with BVVLS affect the functionality of the transporter in human intestinal epithelial cells and that this effect is mediated via alteration in membrane targeting and/or transporter activity. This may inspire us whether C20orf54 missense mutations happened in patients with ESCC, then affects the expression of C20orf54 in ESCC tissues. Huang et al. [20] analyzed the C20orf54 changes in 5 cases of cardiac cancer tissues and the adjacent tissues using exon sequencing, found that two mutation sites (1139C > T and 1172C > C20orf54) of the third exon of gene T can cause the change of amino acids, and this variation may cause abnormal protein expression in cardiac cancer tissues. However, Jiang et al. [11], found no mutations in the ESCC tissues and the adjacent esophageal tissues in 26 ESCC patients with exon sequencing. This discrepancy may result from the tumor type and number of cases, but more studies should be conducted to clarify the mechanism of the inconsistent expression. Gene exon sequencing of riboflavin transporter in cardia cancer and cancerous tissue

The detailed biologic significance of altered C20orf54 expression in cancers remains poorly understood. One of the most important findings of this study was that the low C20orf54 expression in ESCC tissues is associated with poor differentiation, lymph node metastasis, and invasion depth. Our results were consistent with the gastric adenocarcinoma findings of Eli et al. [13], but differ from those of Aili et al. [12], who found that C20orf54 expression in CSCC has no difference with tumor differentiation. However, the mechanism of this phenomenon is still unknown. Several antithetic studies found that the knockdown of C20orf54 caused decreases in the intracellular riboflavin status and inhibitions of cell proliferation and increased the P21 and P27 protein levels, then lead to cell cycle arrest at G1-G1/S [11]. Although contrary to our results, it infers a possible mechanism to explain the said phenomenon. Previous studies show that P21 was correlated with tumor differentiation, invasion depth, and metastasis [21]. These results indicate that C20orf54 can regulate tumor differentiation, lymph node metastasis, and invasion depth through P21, but more experiments should be conducted to explain this hypothesis.

Apart from the genetic effect on ESCC development, tobacco smoking and alcohol consumption are the two main environmental risk factors for ESCC that cover more than 80% of ESCC in developed countries [22]. However, smoking and drinking are the minor risk factors for ESCC in the present study, which is consistent with previously reported results [23]. However, C20orf54 expression can interact with the metabolic changes induced by smoking and drinking, as well as influence the ESCC susceptibility. Therefore, we speculate that the C20orf54 gene change can be the molecular basis of the dietary supplementation of riboflavin in the presence of significant individual differences. However, the exact mechanism should be explored in further experiments.

We formed the ROC curves and AUC to evaluate the specificity and sensitivity of C20orf54 both in ESCC and ESIN. We obtained high sensitivity and specificity values, which could highlight the potential usefulness of C20orf54 as a novel clinical diagnostic biomarker and therapeutic target for ESCC treatment. The sensitivity and specificity of ESIN are inadequate, which can be related to the small sample size. We apply this method in the current study to investigate the C20orf54 expression on the basis of the reported results of immunohistochemistry with high credibility, convenience, stability, and low cost; additional work using other protein analysis techniques is clearly necessary to validate our present results [24–26].

Our results show that ESCC patients with higher C20orf54 expression had a longer postoperative survival time than those with lower C20orf54 expression, but the Cox analysis reveals that C20orf54 defective expression could not serve as a significant independent prognostic factor to predict the risk of death. The relationship between C20orf54 expression and overall survival rates has never been analyzed before. We thus conclude that our study is the first report that demonstrates an association between C20orf54 expression and ESCC prognosis. Investigating other clinical parameters with survival time reveals that lymph node metastasis, TNM stage, tumor location, and invasion depth are significantly different (all *P* < 0.05), whereas differentiation, smoking status, and drinking status have no difference. The discrepancies between these results can be attributed to the diversity in tumor types analyzed, heterogeneity of methods used, and limitations of the small sample sizes. We should expand the sample size and extend the follow-up time to clarify the relationship between the survival time and these parameters.

## Conclusions

This study is the first to show that C20orf54 protein is defectively expressed in ESCC and ESIN tissues and that such low C20orf54 expression is correlated with poor differentiation, invasion depth, and lymph node metastasis of ESCC. We also presented the possible pathway that C20orf54 can involve in the ESCC process. Consequently, the C20orf54 defective expression can be useful in identifying ESCC patients who are likely to experience shorter postoperative survival times. Our data suggest that C20orf54 is useful as a prognostic and diagnostic biomarker and can also be a novel therapeutic target for ESCC. We require more research to validate these concepts and fully elucidate the functional mechanism of this complex protein.

## Data Availability

The datasets used and/or analyzed during the current study are available from the corresponding author on reasonable request.
